# Supplementary material for: A fault-intrusion-tolerant system and deadline-aware algorithm for scheduling scientific workflow in the cloud
Source: PeerJ Comput Sci. 2021 Nov 2;7:e747. doi: 10.7717/peerj-cs.747 (PMC8576554; doi:10.7717/peerj-cs.747)
Supplement: Supplemental Information 6 [file peerj-cs-07-747-s006.zip › Myproject1.1/docs/org/cloudbus/cloudsim/FileAttribute.html]

FileAttribute


JavaScript is disabled on your browser.


- Overview
- Package
- Class
- Use
- Tree
- Deprecated
- Index
- Help

- Prev Class
- Next Class

- Frames
- No Frames

- All Classes

- Summary:
- Nested |
- Field |
- Constr |
- Method

- Detail:
- Field |
- Constr |
- Method


org.cloudbus.cloudsim

## Class FileAttribute

- java.lang.Object
- - org.cloudbus.cloudsim.FileAttribute

- ---

    

  ```
  public class FileAttribute
  extends java.lang.Object
  ```

  A class for storing related information regarding to a `gridsim.datagrid.File` entity.

  Since:
  :   CloudSim Toolkit 1.0

- - ### Constructor Summary

    Constructors

    Constructor and Description

    `FileAttribute(java.lang.String fileName,
    int fileSize)`

    Allocates a new FileAttribute class.
  - ### Method Summary

    Methods

    | Modifier and Type | Method and Description |
    | --- | --- |
    | `boolean` | `copyValue(FileAttribute attr)` Copy the values of this object into another FileAttribute class |
    | `int` | `getAttributeSize()` Gets the size of this object (in byte).  NOTE: This object size is NOT the actual file size. |
    | `int` | `getChecksum()` Gets the file checksum |
    | `double` | `getCost()` Gets the cost associated with this file |
    | `long` | `getCreationTime()` Gets the file creation time (in millisecond) |
    | `int` | `getFileSize()` Gets the file size (in MBytes) |
    | `int` | `getFileSizeInByte()` Gets the file size (in bytes) |
    | `double` | `getLastUpdateTime()` Gets the last update time (in seconds) |
    | `java.lang.String` | `getName()` Returns the file name |
    | `java.lang.String` | `getOwnerName()` Gets the owner name of this file |
    | `int` | `getRegistrationID()` Gets the file registration ID |
    | `int` | `getResourceID()` Gets the resource ID that stores this file |
    | `int` | `getType()` Gets this file type |
    | `boolean` | `isMasterCopy()` Checks whether this file is a master copy or replica |
    | `boolean` | `isReadOnly()` Checks whether this file is a read only or not |
    | `boolean` | `isRegistered()` Checks if this file already registered to a Replica Catalogue |
    | `boolean` | `setChecksum(int checksum)` Sets the checksum of this file |
    | `boolean` | `setCost(double cost)` Sets the cost associated with this file |
    | `boolean` | `setCreationTime(long creationTime)` Sets the file creation time (in millisecond) |
    | `boolean` | `setFileSize(int fileSize)` Sets the file size (in MBytes) |
    | `void` | `setMasterCopy(boolean masterCopy)` Marks this file as a master copy or replica |
    | `void` | `setName(java.lang.String name)` Sets the file name |
    | `boolean` | `setOwnerName(java.lang.String name)` Sets the owner name of this file |
    | `void` | `setReadOnly(boolean readOnly)` Marks this file as a read only or not |
    | `boolean` | `setRegistrationId(int id)` Sets the file registration ID (published by a Replica Catalogue entity) |
    | `boolean` | `setResourceID(int resourceID)` Sets the resource ID that stores this file |
    | `boolean` | `setType(int type)` Sets the file type (e.g. |
    | `boolean` | `setUpdateTime(double time)` Sets the last update time of this file (in seconds)  NOTE: This time is relative to the start time. |

    - ### Methods inherited from class java.lang.Object

      `clone, equals, finalize, getClass, hashCode, notify, notifyAll, toString, wait, wait, wait`

- - ### Constructor Detail


    - #### FileAttribute

      ```
      public FileAttribute(java.lang.String fileName,
                   int fileSize)
                    throws ParameterException
      ```

      Allocates a new FileAttribute class.

      Parameters:
      :   `fileName` - file name
      :   `fileSize` - size of this file (in bytes)

      Throws:
      :   `ParameterException` - This happens when one of the following scenarios occur:
          - the file name is empty or null- the file size is zero or negative numbers
  - ### Method Detail


    - #### copyValue

      ```
      public boolean copyValue(FileAttribute attr)
      ```

      Copy the values of this object into another FileAttribute class

      Parameters:
      :   `attr` - a FileAttribute object (the destination)

      Returns:
      :   true if the copy operation is successful, false otherwise


    - #### setCreationTime

      ```
      public boolean setCreationTime(long creationTime)
      ```

      Sets the file creation time (in millisecond)

      Parameters:
      :   `creationTime` - the file creation time (in millisecond)

      Returns:
      :   true if successful, false otherwise


    - #### getCreationTime

      ```
      public long getCreationTime()
      ```

      Gets the file creation time (in millisecond)

      Returns:
      :   the file creation time (in millisecond)


    - #### setResourceID

      ```
      public boolean setResourceID(int resourceID)
      ```

      Sets the resource ID that stores this file

      Parameters:
      :   `resourceID` - a resource ID

      Returns:
      :   true if successful, false otherwise


    - #### getResourceID

      ```
      public int getResourceID()
      ```

      Gets the resource ID that stores this file

      Returns:
      :   the resource ID


    - #### setOwnerName

      ```
      public boolean setOwnerName(java.lang.String name)
      ```

      Sets the owner name of this file

      Parameters:
      :   `name` - the owner name

      Returns:
      :   true if successful, false otherwise


    - #### getOwnerName

      ```
      public java.lang.String getOwnerName()
      ```

      Gets the owner name of this file

      Returns:
      :   the owner name or null if empty


    - #### getAttributeSize

      ```
      public int getAttributeSize()
      ```

      Gets the size of this object (in byte).  
      NOTE: This object size is NOT the actual file size. Moreover, this size is used for
      transferring this object over a network.

      Returns:
      :   the object size (in byte)


    - #### setFileSize

      ```
      public boolean setFileSize(int fileSize)
      ```

      Sets the file size (in MBytes)

      Parameters:
      :   `fileSize` - the file size (in MBytes)

      Returns:
      :   true if successful, false otherwise


    - #### getFileSize

      ```
      public int getFileSize()
      ```

      Gets the file size (in MBytes)

      Returns:
      :   the file size (in MBytes)


    - #### getFileSizeInByte

      ```
      public int getFileSizeInByte()
      ```

      Gets the file size (in bytes)

      Returns:
      :   the file size (in bytes)


    - #### setUpdateTime

      ```
      public boolean setUpdateTime(double time)
      ```

      Sets the last update time of this file (in seconds)  
      NOTE: This time is relative to the start time. Preferably use
      `gridsim.CloudSim#clock()` method.

      Parameters:
      :   `time` - the last update time (in seconds)

      Returns:
      :   true if successful, false otherwise


    - #### getLastUpdateTime

      ```
      public double getLastUpdateTime()
      ```

      Gets the last update time (in seconds)

      Returns:
      :   the last update time (in seconds)


    - #### setRegistrationId

      ```
      public boolean setRegistrationId(int id)
      ```

      Sets the file registration ID (published by a Replica Catalogue entity)

      Parameters:
      :   `id` - registration ID

      Returns:
      :   true if successful, false otherwise


    - #### getRegistrationID

      ```
      public int getRegistrationID()
      ```

      Gets the file registration ID

      Returns:
      :   registration ID


    - #### setType

      ```
      public boolean setType(int type)
      ```

      Sets the file type (e.g. raw, tag, etc)

      Parameters:
      :   `type` - a file type

      Returns:
      :   true if successful, false otherwise


    - #### getType

      ```
      public int getType()
      ```

      Gets this file type

      Returns:
      :   file type


    - #### setChecksum

      ```
      public boolean setChecksum(int checksum)
      ```

      Sets the checksum of this file

      Parameters:
      :   `checksum` - the checksum of this file

      Returns:
      :   true if successful, false otherwise


    - #### getChecksum

      ```
      public int getChecksum()
      ```

      Gets the file checksum

      Returns:
      :   file checksum


    - #### setCost

      ```
      public boolean setCost(double cost)
      ```

      Sets the cost associated with this file

      Parameters:
      :   `cost` - cost of this file

      Returns:
      :   true if successful, false otherwise


    - #### getCost

      ```
      public double getCost()
      ```

      Gets the cost associated with this file

      Returns:
      :   the cost of this file


    - #### isRegistered

      ```
      public boolean isRegistered()
      ```

      Checks if this file already registered to a Replica Catalogue

      Returns:
      :   true if it is registered, false otherwise


    - #### setMasterCopy

      ```
      public void setMasterCopy(boolean masterCopy)
      ```

      Marks this file as a master copy or replica

      Parameters:
      :   `masterCopy` - a flag denotes true for master copy or false for a
          replica


    - #### isMasterCopy

      ```
      public boolean isMasterCopy()
      ```

      Checks whether this file is a master copy or replica

      Returns:
      :   true if it is a master copy or false otherwise


    - #### setReadOnly

      ```
      public void setReadOnly(boolean readOnly)
      ```

      Marks this file as a read only or not

      Parameters:
      :   `readOnly` - a flag denotes true for read only or false for re-writeable


    - #### isReadOnly

      ```
      public boolean isReadOnly()
      ```

      Checks whether this file is a read only or not

      Returns:
      :   true if it is a read only or false otherwise


    - #### setName

      ```
      public void setName(java.lang.String name)
      ```

      Sets the file name

      Parameters:
      :   `name` - the file name


    - #### getName

      ```
      public java.lang.String getName()
      ```

      Returns the file name

      Returns:
      :   the file name


- Overview
- Package
- Class
- Use
- Tree
- Deprecated
- Index
- Help

- Prev Class
- Next Class

- Frames
- No Frames

- All Classes

- Summary:
- Nested |
- Field |
- Constr |
- Method

- Detail:
- Field |
- Constr |
- Method
